# Supplementary material for: VIBESegmentator: full body MRI segmentation for the NAKO and UK Biobank
Source: Eur Radiol. 2025 Oct 9;36(4):2548–62. doi: 10.1007/s00330-025-12035-9 (PMC13035649; doi:10.1007/s00330-025-12035-9)
Supplement: Supplementary file 1 — ELECTRONIC SUPPLEMENTARY MATERIAL [file 330_2025_12035_MOESM1_ESM.pdf]

VIBESegmentator: Full Body MRI Segmentation for the NAKO and UK Biobank

ELECTRONIC SUPPLEMENTARY MATERIAL

1. Intermediate Failure Analysis

We used the third last iteration and the abdominal segmentation network by Kart et al.[1, 2] to find failure cases to include them in the training. We segmented 1,000 images (448 female, mean age 50) using the abdominal segmentation network[1, 2] and compared our second last model's output. We inspected 40 outliers where at least one segmentation was two standard deviations below the mean Dice score. Four images had a water/fat inversion error in the MR signal. Our segmentation was robust to these errors, producing only minor inaccuracies at the inversion lines. In addition to these issues, both models produced errors for horseshoe kidneys, liver and kidney cysts, and abnormally large kidneys and spleens, which were only partially segmented. Our model produced faults in 12 out of 40 cases, while the abdominal segmentation method[1, 2] produced faults in 35 out of 40 cases. We counted a segmentation as faulty if at least 10% of the organ was missing or over-segmented. We corrected these and added them to the next training iteration. We repeated the experiment with another 1,000 images (421 female, mean age 50). Results showed that the model was more robust to cyst and size changes, and only extreme cases caused segmentation errors after including other images with pathologies. We did not find issues with our pancreas segmentation in both tests, but observed that the abdominal segmentation network[1, 2] has issues with pancreas segmentation if the person has larger amounts of body fat. Known failure cases include subjects with additional or displaced organs, such as with kidneys, or unseen pathologies. Similar issues occur with the head, feet, hands, and forearms, as these are outside the field of view of our training data. We have not observed segmentation errors in bone and lung segmentation in our healthy subjects so far.

2. References

1. Kart T, Fischer M, Winzeck S, et al (2022) Automated imaging-based abdominal organ segmentation and quality control in 20,000 participants of the UK Biobank and German National Cohort Studies. Scientific Reports 12:18733

2. Kart T, Fischer M, Küstner T, et al (2021) Deep learning-based automated abdominal organ segmentation in the UK Biobank and German National Cohort Magnetic Resonance Imaging Studies. Investigative Radiology 56:401–408
